# Tribological Performance of Additively Manufactured AISI H13 Steel in Different Surface Conditions

**DOI:** 10.3390/ma14040928

**Published:** 2021-02-16

**Authors:** Elisabeth Guenther, Moritz Kahlert, Malte Vollmer, Thomas Niendorf, Christian Greiner

**Affiliations:** 1 Karlsruhe Institute of Technology (KIT), Institute for Applied Materials (IAM), Kaiserstrasse 12, 76131 Karlsruhe, Germany; eli.guenther@gmx.de; 2 KIT IAM-CMS MicroTribology Center (µTC), Strasse am Forum 5, 76131 Karlsruhe, Germany; 3 Institute of Materials Engineering, Universität Kassel, Mönchebergstraße 3, 34125 Kassel, Germany; kahlert@uni-kassel.de (M.K.); vollmer@uni-kassel.de (M.V.); niendorf@uni-kassel.de (T.N.)

**Keywords:** tribology, additive manufacturing, selective laser melting, surface roughness

## Abstract

Additive manufacturing of metallic tribological components offers unprecedented degrees of freedom, but the surface roughness of most as-printed surfaces impedes the direct applicability of such structures, and postprocessing is necessary. Here, the tribological performance of AISI H13 steel samples was studied. These were additively manufactured through laser powder bed fusion (L-PBF), also referred to as selective laser melting (SLM). Samples were tested in four different surface conditions: as-printed, polished, ground and polished, and laser-surface-textured (LST) with round dimples. Friction experiments were conducted in a pin-on-disk fashion against bearing steel disks under lubrication with an additive-free mineral base oil for sliding speeds between 20 and 170 mm/s. Results demonstrated that, among the four surface treatments, grinding and polishing resulted in the lowest friction coefficient, followed by the as-printed state, while both polishing alone and laser-surface texturing increased the friction coefficient. Surprisingly, direct correlation between surface roughness and friction coefficient, i.e., the rougher the surface was, the higher the friction force, was not observed. Wear was minimal in all cases and below what could be detected by gravimetrical means. These results highlight the need for an adequate post-processing treatment of additively manufactured parts that are to be employed in tribological systems.

## 1. Introduction

The global need to conserve resources and energy has several consequences. For one, it is desirable to reduce waste during the manufacturing of components; second, the lifetime and energy necessary to operate a component or system ought to be optimized. The first aspect can be addressed through the near net-shape manufacturing of components by 3D-printing, i.e., additive manufacturing (AM), and through the high degree of geometric freedom provided by respective techniques, eventually leading to additional weight reduction, e.g., through topology optimization [1,2,3]. The second aspect can be approached by better tribological practices. This is true as many engineering systems contain moving parts and are thereby susceptible to friction and wear. Tribological losses are responsible for more than 20% of the global primary energy usage [4]. It is, therefore, obvious that the additive manufacturing of tribological components with superior friction and wear properties may hold significant potential to save precious resources and energy. At the same time, realizing this potential is seriously impeded mainly due to the high surface roughness of as-printed metallic parts produced by additive manufacturing [5,6,7].

In this context, the literature mainly investigated parts that were manufactured by laser powder bed fusion (L-PBF), also referred to as selective laser melting (SLM) [8,9,10,11,12,13]. Using this method, Al–Si and Ti6Al4V alloys, and 316L steel were printed and tested with or without further changes to the surfaces themselves [8,9,10,11,12,13]. Moreover, hybrid additive manufacturing methods consisting of laser cladding, milling, ultrasonic surface rolling [14], and different coating systems [15,16] were used to improve the tribological performance of additively manufactured materials. The authors of these studies were able to show the possibility of using the SLM process for directly printing coating material onto additively manufactured substrate materials, which opens up a wide field of new applications. However, the present study focuses on the tribological performance of additively manufactured steel. Bajaj et al. [1] reported that there was no conclusive available information on the wear performance of SLM-produced AISI H13. Investigations on tool steel [17] and high-speed steel [18] revealed the tendency of the wear rate, i.e., the removed volume per sliding distance, to decrease with the increased hardness of the material, resulting in increased wear resistance of additively manufactured parts.

When comparing the tribological performance of 316L stainless-steel samples that were processed by SLM to traditional processes, both sets of samples were polished to the same level of surface roughness [9]. These experiments found that SLM parts performed either better or worse than traditionally manufactured ones did, depending on the counter body material. In another study, 316L surfaces were again ground and polished, and the effect of different build-up directions was investigated [13]. When purposefully printing stainless-steel parts with a varying degree of porosity, due to these, the friction coefficient in the hydrodynamic branch of the Stribeck curve [19] decreased compared to that in fully dense samples [11]. To achieve this effect, samples were not used as printed, but polished manually to a root-mean-square (RMS) roughness of 100 nm [11]. However, such post-processing could impede the usefulness of additive manufacturing. The need to alter the as-printed surface was also addressed for Ti6Al4V. Again, samples were polished after 3D-printing and subjected to an oxidation treatment to improve their wear resistance [12]. Interestingly, the surface roughness inherent to additive manufacturing in this case was used as a means to study the polishing processes of rough surfaces [20]. Grinding and polishing was also necessary when a CoCrFeMnNi high-entropy alloy was processed via an additive manufacturing route, and tribologically tested [21].

Selective laser melting was also employed to create ceramic coatings by additive manufacturing, such as ZrB_2_, ZrC, B_4_C [15], a NiCrAlY bond coat [16], and a nickel-based superalloy [22], to name a few examples. These coatings can also increase wear resistance.

This brief and by no means comprehensive overview over some of the existing literature demonstrates in almost all cases that, when the tribological performance of parts processed via additive manufacturing was assessed, the samples were ground and polished before they were subjected to the tribological load. However, what was not answered so far was how much of an effect this smoothing of the as-printed samples had on friction forces, or whether it was necessary at all. As the strategic morphological texturing of surfaces is a prolific field of tribological research [23,24,25], a certain amount of surface roughness could be beneficial to friction and wear. Therefore, in the present study H13 steel was processed using SLM and then tested to assess friction performance in four different surface states: as-printed, ground, ground and polished, and deliberately laser-surface-textured (LST) with round dimples. Friction forces were measured in a lubricated pin-on-disk contact against bearing steel disks, mainly in the mixed lubrication part of the Stribeck curve. These results are intended to identify how much difference in terms of friction coefficient these four post-processing treatments provoke. These first experiments will hopefully inspire other, more in-depth experimental and modeling research into this important topic, ultimately allowing the strategic 3D-printing of metals for tribological components with the least amount of post-processing necessary.

## 2. Materials and Methods

### 2.1. Additive Manufactoring

**Figure 1:**
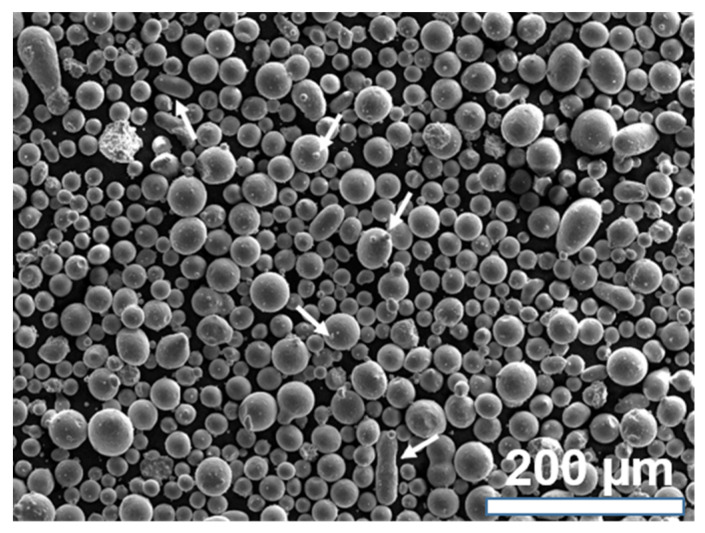
Characteristic secondary-electron (SE) micrograph of AISI H13 powder used in the present study. Some satellites and oval particles are highlighted with white arrows.

AISI H13 samples were manufactured using gas atomized powder with particle size between 18 and 44 µm, and a nominal chemical composition of 0.4% C, 5.3% Cr, 1.4% Mo, 1% V (wt %). Figure 1 shows a characteristic secondary-electron (SE) micrograph of the powder, revealing homogeneous morphology, and only a few satellites and oval particles, highlighted with white arrows. For selective laser melting, an SLM 280^HL^ (SLM Solutions Group AG, Lubeck, Germany) equipped with a high-temperature substrate-plate heating device was used. Cuboids with dimensions of 6 (width) × 40 (depth) × 40 mm (height) were built upright on the substrate plate, which was heated up to 200 °C during processing. One of the long axes was oriented along the build direction (BD) during SLM. Different process parameters were used for contour and volume (Table 1).

An exposure strategy with a maximal scan-track length of 10 mm was chosen to melt the volume. Scan-track directions were rotated by 33° every layer. Afterwards, samples were mechanically released from the substrate plate.

### 2.2. Surface Treatment and Characterization

From the cuboids printed through the SLM process described above, cylindrical samples of 8 mm in diameter and thickness of 5 mm were manufactured through electrical discharge machining, with their symmetry axis oriented perpendicular to the BD. For the as-printed samples, no further surface treatment was performed. Before and after the experiments, all samples were stored in a desiccator under a high vacuum.

For the polishing surface treatment, the as-built surfaces of the samples were manually polished only with monocrystalline diamond suspensions of 3 and 1 µm in particle size (Strikers, Willich, Germany) for about 10 min each. Between polishing steps, samples were cleaned in isopropanol under ultrasound for 5 min.

“Ground and polished” samples were manually ground with SiC papers of mesh P220, P600, and P1200. This was followed by mechanical polishing on synthetic silk clothes (provided by Cloeren Technology GmbH, Wegberg, Germany) using monocrystalline diamond suspensions with particle sizes of 3 and 1 µm (Struers, Willich, Germany) for about 10 min each. Between grinding and polishing steps, samples were sonicated in isopropanol for 5 min in order to remove debris.

**Figure 2:**
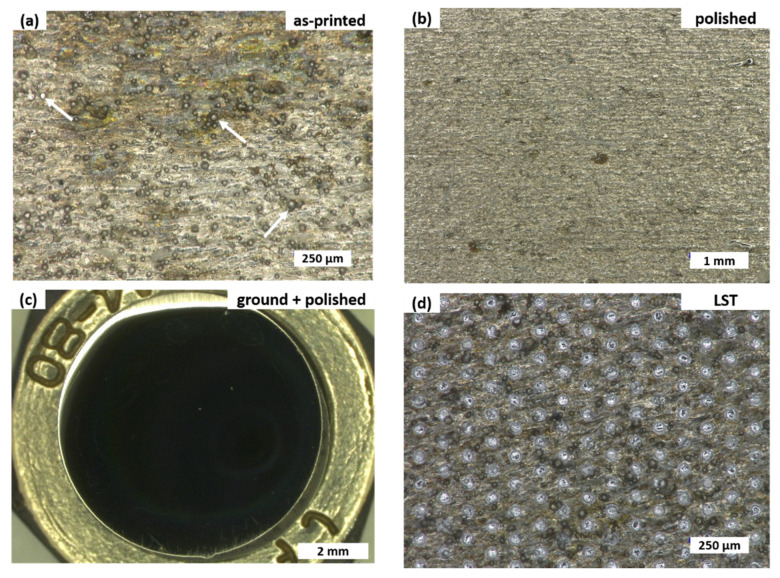
Optical micrographs before tribological tests. (**a**) As-printed, (**b**) polished, (**c**) ground and polished, and (**d**) laser-surface-textured. H13 steel samples initially were prepared by selective laser melting (SLM). Magnification for each image chosen such to best represent samples. Unmelted powder in (**a**) is highlighted by white arrows.

Before the tribological experiments, each sample was sonicated for 15 min more in isopropanol. Before and after every tribological experiment, sample surfaces were documented through optical micrographs taken with a VHX-600 digital optical microscope (Keyence, Osaka, Japan). Surface roughness was also measured with an optical profilometer (Sensofar PluNeox, Barcelona, Spain) before and after each experiment. Exemplary micrographs for all four surface treatments before the tribological tests are presented in Figure 2.

### 2.3. Tribological Testing

Tribological experiments were conducted with a laboratory pin-on-disk tribometer from CSM Instruments (CSM Instruments, Peseux, Switzerland). Bearing steel 100Cr6 (AISI 5210, EisenSchmitt, Karlsruhe, Germany) disks with a diameter of 70 mm were used as counter bodies. Discs were hardened and tempered to Vickers hardness of about 800 HV. Hardness was quantified with a Q10A+ Vickers hardness tester (Qness GmbH, Golling, Austria). Disks were ground to R_a_ roughness of 0.1 µm, measured by a Hommelwave Waveline W800 (Jenoptic AG, Jena, Germany) tactile profilometer. Waviness along the radius where the tribological experiments were conducted was below 1 µm, as determined with a Sensofar PluNeox white light interferometer (Sensofar, Barcelona, Spain). Additively manufactured H13 steel pins with a diameter of 8 mm were mounted in self-aligning holders. Once the pin had been mounted, it was placed on the disk and glued into the holder with a two-component epoxy (UHU Plus Sofortfest, Buehl, Germany), with the normal load of 10 N already applied via dead weights. This process ensured a proper flat-on-flat contact. The epoxy was hardened for 15 min before 3 mL of FVA-2, a mineral base oil without additives, was added to allow for ample lubrication. At room temperature, this oil had a dynamic viscosity of approximately 80 mPa·s. For the tribological experiments, a characteristic speed profile was tested, starting at a sliding speed of 170 mm/s, which was reduced to 20 mm/s in 9 separate steps (100, 80, 70, 60, 50, 40, and 30 mm/s). Each sliding speed was held for five minutes. This speed ramp was repeated 5 times. All presented friction data are the result of averaging these 5 ramps and at least 2 individual experiments. These sliding speeds were chosen to mainly test in the mixed lubrication regime and the transition to hydrodynamic lubrication, respectively. Ambient temperature during the experiments was 23 ± 2 °C. Relative humidity was controlled to 40 ± 3% by enclosing the setup in a Plexiglas box where humidity was equilibrated for at least 1 hour prior to every experiment.

### 2.4. Laser Surface Texturing

Laser surface texturing (LST) was carried out directly on the as-printed surface of the cylindrical H13 steel samples with a diameter of 8 mm and a thickness of 5 mm, which were cut from the as-printed cuboids by electrical discharge machining. A Q-switched Ytterbium-doped fiber laser, Piranha II (Acsys Instruments, Kornwestheim, Germany), was employed. The wavelength was 1064 nm, and the laser source power was set to 11.2 W. Samples were textured with circular dimples of 40 µm in diameter and a packing density of 10%. Dimple depth was kept constant at 4 µm. In previous experiments, this texture was proven to be superior, especially in mixed lubrication where a reduction in friction forces of up to 80% was found [27,28]. The debris that formed during laser ablation was not removed by an additional polishing step (see Figure 2d).

## 3. Results and Discussion

H13 steel was successfully processed by SLM, following process parameters already detailed in previous work [26]. The final density of the steel cuboids was above 99%. After the electrical discharge machining of cylinders for tribological experiments, samples were either left as-printed, solely polished, ground and polished, or laser-surface-textured with round dimples. Each of these surface treatments had an effect on surface roughness, with the resulting average area surface roughness (S_a_) values summarized in Table 2.

The roughness data presented in Table 2 were not entirely as expected. First, an average areal surface roughness S_a_ in the range of 3 to 6 μm is quite rough. Second, it is surprising that surface roughness decreased upon laser surface texturing. The optical micrographs presented in Figure 2 support the interferometry results. Most probably the as-printed surface was so rough that LST decreased roughness due to partial remelting of the surface. One can also note that only polishing the as-printed surface did not change the roughness significantly. This is due to the fact that the surface roughness upon SLM was influenced by many different factors contributing to the final appearance of the as-built surface [29]. Two of the most relevant aspects were partially melted powder particles stuck to the surface (highlighted by arrows in Figure 2a) and general process inherent characteristics based on the layer-wise manufacturing principle and local melt-pool dynamics. Pure polishing was not able to fully flatten the surface, as the material removal rate was not sufficient to remove the roughness due to the inherent characteristics of SLM, detailed above. Furthermore, some remaining powder particles can be seen in Figure 2b. Thus, roughness was still fairly pronounced in the polished condition, and the S_a_ value was hardly affected (Table 2).

**Figure 3:**
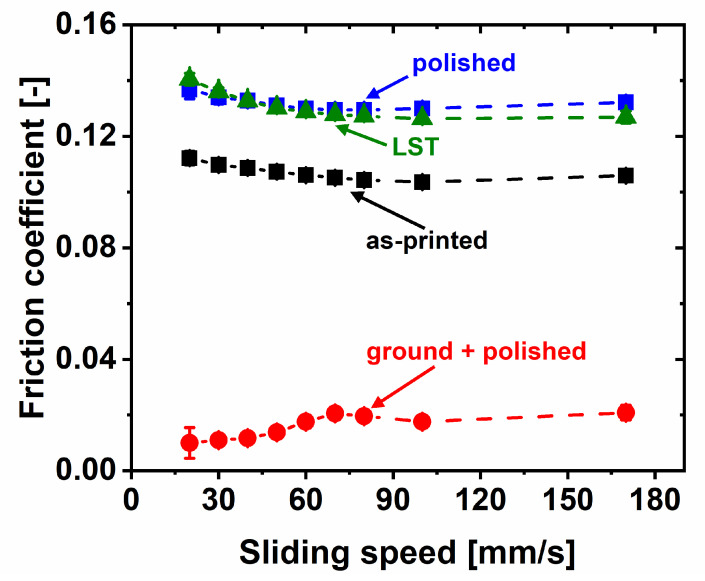
Average friction coefficient as function of sliding speed for as-printed, polished, ground and polished, and laser-surface-textured samples. Experiments were performed in a pin-on-disk fashion against 100Cr6 bearing steel disks. Sliding speed was varied between 20 and 170 mm/s, and ramps were repeated five times. Results are the average of these five ramps deduced from two independent experiments. For most data points, error bars representing standard deviation are smaller than the size of the symbols. Normal force was 10 N. FVA2 unadditivated mineral oil was used to lubricate the contact.

The frictional behavior of these four surface conditions was tested in lubricated pin-on-disk contacts against bearing steel counter bodies. The resulting average friction coefficients for experiments run with sliding speeds between 20 and 170 mm/s are presented in Figure 3.

When comparing the data visualized in Figure 3, grinding and polishing was extremely effective in reducing friction compared to the as-printed state. While samples that were tested directly after the additive manufacturing process without any further processing exhibited a minimal friction coefficient of about 0.1, grinding and polishing reduced this value by one order of magnitude to 0.01. In addition, grinding and polishing kept the tribological contact in the hydrodynamic regime for all tested sliding speeds. For the as-printed sample, the transition between hydrodynamic and mixed lubrication took place at a speed of 100 mm/s. When altering the surface morphology, e.g., through laser texturing, it is a common goal to shift this transition to smaller sliding speeds, as less wear is expected in the hydrodynamic regime since both sliding partners are fully separated by an oil film [23,27].

When comparing the as-printed to the polished and laser-surface-textured samples, it becomes apparent that neither polishing nor laser texturing improved the frictional characteristics. Furthermore, both increased the minimal friction force by about 30%, from µ = 0.10 for the as-printed state to µ = 0.13 for both the polished and the laser-surface-textured samples. This was surprising, as the chosen texturing parameters of a dimple diameter of 40 µm, a packing density of 10%, and a dimple aspect ratio of 0.1 proved successful in the past, where friction reductions of up to 80% were possible [27,30]. While these experiments had all been carried out with polished samples, and friction reduction was mainly attributed to additional hydrodynamic lift due to the surface textures [31], at least some positive effect was expected in the current case of the rough additively manufactured samples. The fact that experimental data show the opposite most probably demonstrates that the effects related to the initial surface-roughness (in the as-built surface condition) predominated over the potential benefits from LST.

There is only a very rough correlation between friction results and surface roughness. The ground and polished samples had about an order of magnitude lower S_a_ roughness values compared to those of the other surface conditions. In line with this difference, a friction coefficient approximately one order of magnitude lower was determined. When investigating this correlation in more detail, it brakes down. For example, the laser-surface-textured samples for both before and after the tribological tests had a 20 to 30% smaller surface roughness compared to the as-printed ones. The friction coefficient for the LST samples might, therefore, be smaller compared to that of the as-printed state; this was the reasoning behind laser texturing in the first place. However, experimental results were exactly the opposite. The LST samples are characterized by a minimal friction coefficient, which is a factor of 1.3 larger compared to that of the as-printed ones.

Generally, finding a correlation between surface roughness and friction coefficient has proven extremely difficult in the literature. The friction coefficient has a multitude of factors it depends on and singling out an individual one as being decisive has to be considered with caution [32]. According to Blau, it is difficult to estimate which factors have the greatest influence on friction [32]. Friction force *F* can be described as $F=\overset{n}{\underset{i=1}{\sum }}f_{i}$, where *f_i_* represents the partial forces that are responsible for the experimentally measured friction force in a combined fashion. Such partial forces are, for example, the force required to shear the lubricant, to deform adhesive junctions, and to plough hard particles through the surface of a softer material [32,33]. The different surface roughness and hardness of a material in a tribological contact may, therefore, influence some or even all of the individual force components, and result in a complex interplay between them. This might be one of the reasons why a straightforward correlation between surface parameters, such as surface roughness, and friction forces is very difficult to establish, and current attempts give somewhat contradictory results. For seals and seal-like structures, especially in the context of laser surface texturing, a positive effect of surface roughness on their performance was reported [34]. The same is true for the effect that surface roughness might have in hydrodynamic lubrication, where additional hydrodynamic lift was associated with surface roughness [35,36]. This is in contradiction to recent data, other modeling, and experimental results [34,37].

To gain further understanding of how the roughness profile influences tribological performance, especially in partially lubricated contacts as they are investigated here, fluid dynamics models—as they were developed by Patir and Cheng and extended by Tripp—might be helpful [38,39,40]. Results from their models suggest that the load-bearing capacity of a rough surface cannot be predicted in a straightforward fashion. Moreover, actual properties depend on the exact nature of the surface features making up the roughness. Thus, these features need to be modeled in detail [39].

**Figure 4:**
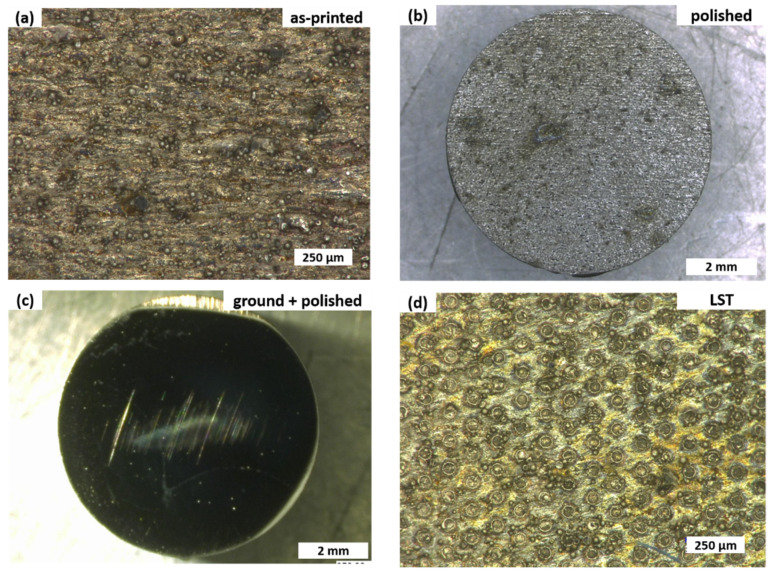
Optical micrographs for samples after tribological experiments. (**a**) As-printed, (**b**) polished, (**c**) ground and polished, and (**d**) laser-surface-textured H13 steel samples initially prepared by SLM. Magnification for each image was chosen as such to best represent the samples.

Future experiments have to consider the effect of the surface treatments considered here on the subsurface microstructure. Following Bowden and Tabor, the plastic behavior of the softer material in a metallic sliding contact is decisive for the friction forces [41,42]. Each metallographic treatment of a surface leads to a so-called Beilby layer [43] that usually consists of smaller grains compared to the original bulk material. Similarly, laser texturing often results in a heat-affected zone of the altered microstructure. These changes in grain size and possibly surface chemistry directly induced by grinding, polishing, and laser texturing could have significant influence on the final friction characteristics, in addition to the surface roughness studied here. Investigating these effects was, however, beyond the scope of the present study. The attempt to quantify wear by gravimetrical means, determining the mass of each sample before and after the tribological experiment, did not give any indication of wear. Even in the region of 1 μg, no change in mass could be determined. This might be taken as a first indication that wear was minimal. Characteristic optical micrographs for each surface treatment after the tribological experiments are presented in Figure 4.

These images second the gravimetrical results. Only very few signs of wear are visible, with the exception of the ground and polished sample (Figure 4c). Here, scratches can be seen, but the material loss associated with them was still too small to make a detectable gravimetrical difference. More detailed wear studies are needed before a conclusive statement about this important aspect of tribological performance will be possible.

## 4. Conclusions

The tribological properties of additively manufactured H13 steel samples in four different surface conditions were tested. Samples were investigated in as-printed, solely polished, ground and polished, or laser-surface-textured states, the latter being characterized by round dimples of 40 µm in diameter. Tribological experiments were run against bearing steel disks in a unidirectional pin-on-disk tribometer. The contact was lubricated with a base oil without additives. The sliding speed was varied between 20 and 170 mm/s to measure the part of the Stribeck curve where the transition between mixed and hydrodynamic lubrication was expected. Tribological data demonstrate that grinding and polishing the as-printed surfaces to a mirror finish of S_a_ = 0.15 µm lowered the friction coefficient by about one order of magnitude compared to the as-printed samples. The ground-and-polished samples were the only ones, where hydrodynamic lubrication was present for all sliding speeds. The minimal friction coefficient for the as-printed state was 0.1, and 0.01 for the ground-and-polished surface state. Contrary to expectation, polishing and laser surface texturing both increased the minimal friction coefficient to about 0.13. There was no clear correlation of these results with surface roughness. Polishing did not alter the S_a_ value compared to the as-printed surface, while laser texturing reduced it. Wear was below the detection limit of the employed gravimetrical method. In the optical micrographs, only few signs of wear events are observed. Detailed fluid dynamics modeling is most probably required to fully understand these results. However, they highlight how strong the effect of post-processing can be. A much better understanding of these processes on the tribological performance of additively manufactured parts used in tribological contacts needs to be established.

## Figures and Tables

**Table 1:** Process parameters used for manufacturing AISI H13 samples [26].

Area	Power, W	Velocity, mm/s	Hatch Distance, mm	Layer Thickness, µm
Volume	270	700	0.12	30
Contour	100	400	-	30

**Table 2:** Average area surface roughness S_a_ of additively manufactured H13 steel samples in surface conditions as-printed, polished, ground and polished, and laser-surface-textured before and after tribological testing. Surface roughness was determined by white-light interferometry at five different positions on each sample. The given error is the standard deviation of these measurements.

Unit	As-Printed	Polished	Ground and Polished	Laser-Textured
**S_a,before_** **(µm)**	5.74 ± 0.40	5.64 ± 0.30	0.15 ± 0.05	3.80 ± 0.90
**S_a,after_** **(µm)**	6.70 ± 0.30	6.10 ± 0.40	0.06 ± 0.01	5.3 ± 0.60

## Data Availability

Raw data and images are available from the corresponding author upon request and have been published under https://doi.org/10.5445/IR/1000129689.
